# Extension and Severity of Self-Reported Side Effects of Seven COVID-19 Vaccines in Mexican Population

**DOI:** 10.3389/fpubh.2022.834744

**Published:** 2022-03-14

**Authors:** María Elena Camacho Moll, Ana María Salinas Martínez, Benjamín Tovar Cisneros, Juan Ignacio García Onofre, Gloria Navarrete Floriano, Mario Bermúdez de León

**Affiliations:** ^1^Department of Molecular Biology, Northeast Biomedical Research Center, Mexican Institute of Social Security, Monterrey, Mexico; ^2^Health Sciences Division, Center for Molecular Diagnosis and Personalized Medicine, Universidad de Monterrey, Monterrey, Mexico; ^3^Epidemiologic and Health Services Research Unit, Mexican Institute of Social Security, Monterrey, Mexico; ^4^School of Public Health and Nutrition, Autonomous University of Nuevo Leon, Monterrey, Mexico; ^5^School of Biological Sciences, Autonomous University of Nuevo Leon, Monterrey, Mexico; ^6^Family Medicine Unit No. 64, Mexican Institute of Social Security, Monterrey, Mexico

**Keywords:** COVID-19, SARS-CoV-2, side effects, local effects, systemic effects, vaccination, vaccine

## Abstract

A few studies examined the comparative side effects of Coronavirus Disease-19 (COVID-19) vaccines. We compared the extension and severity of self-reported side effects of seven COVID-19 vaccines [BNT162b2 (Pfizer-BioNTech), ChAdOx1 (AstraZeneca), mRNA-1273 (Moderna), CoronaVac (Sinovac Life Sciences), Gam-COVID-Vac (Gamaleya's Sputnik V), Ad5-nCoV (CanSinoBIO), and Ad26.CoV2.S (Johnson & Johnson/Janssen)] in the Mexican population. We also evaluated the association of type of vaccine, sex, age, comorbidity, and history of allergies to the extent and severity of side effects. This was a cross-sectional study carried out online between August 12 and September 3, 2021 in Mexico. The first inclusion criterion was to receive a COVID-19 vaccine and the second, being at least 18 years old. The survey link was distributed *via* multiple social media platforms. We questioned about the type of vaccine and symptoms based on short-term side effects reported in the literature. Side effect extension was classified as local, systemic, or both. We asked about the need to take medicine, stop activities/miss work, or seek medical attention. Then, a severity index was constructed based on responses. Descriptive and stepwise multivariate logistic ordinal regression analyses were used to calculate odds ratio (OR) and 95% CI for each outcome adjusted by potential confounders. The mean age was 38.9 ± 11.0 years (*n* = 4,024). Prevalence of at least one side effect varied between vaccines and by a number of doses. At dose 1, ChAdOx1 was the vaccine with the highest rate of at least one side effect (85%) followed by Gam-COVID-Vac (80%). Both were associated to greater extension (adjusted OR 2.53, 95% CI 2.16, 2.96 and adjusted OR 2.41, 95% CI 1.76, 3.29, respectively) and severity of side effects (adjusted OR 4.32, 95% CI 3.73, 5.00 and adjusted OR 3.00, 95% CI 2.28, 3.94, respectively). Young age (<50 years), female sex, comorbidity, and history of allergies were associated with greater extension and severity, independent of the type of vaccine and potential confounders. At dose 2, mRNA-1273 was the vaccine with the highest rate of side effects (88%) and the only vaccine associated to greater extension (adjusted OR 2.88, 95% CI 1.59, 5.21) and severity of symptoms (adjusted OR 3.14, 95% CI 1.82, 5.43). Continuous studies are necessary to acknowledge more post-vaccine symptoms in different populations.

## Introduction

**Figure 1:**
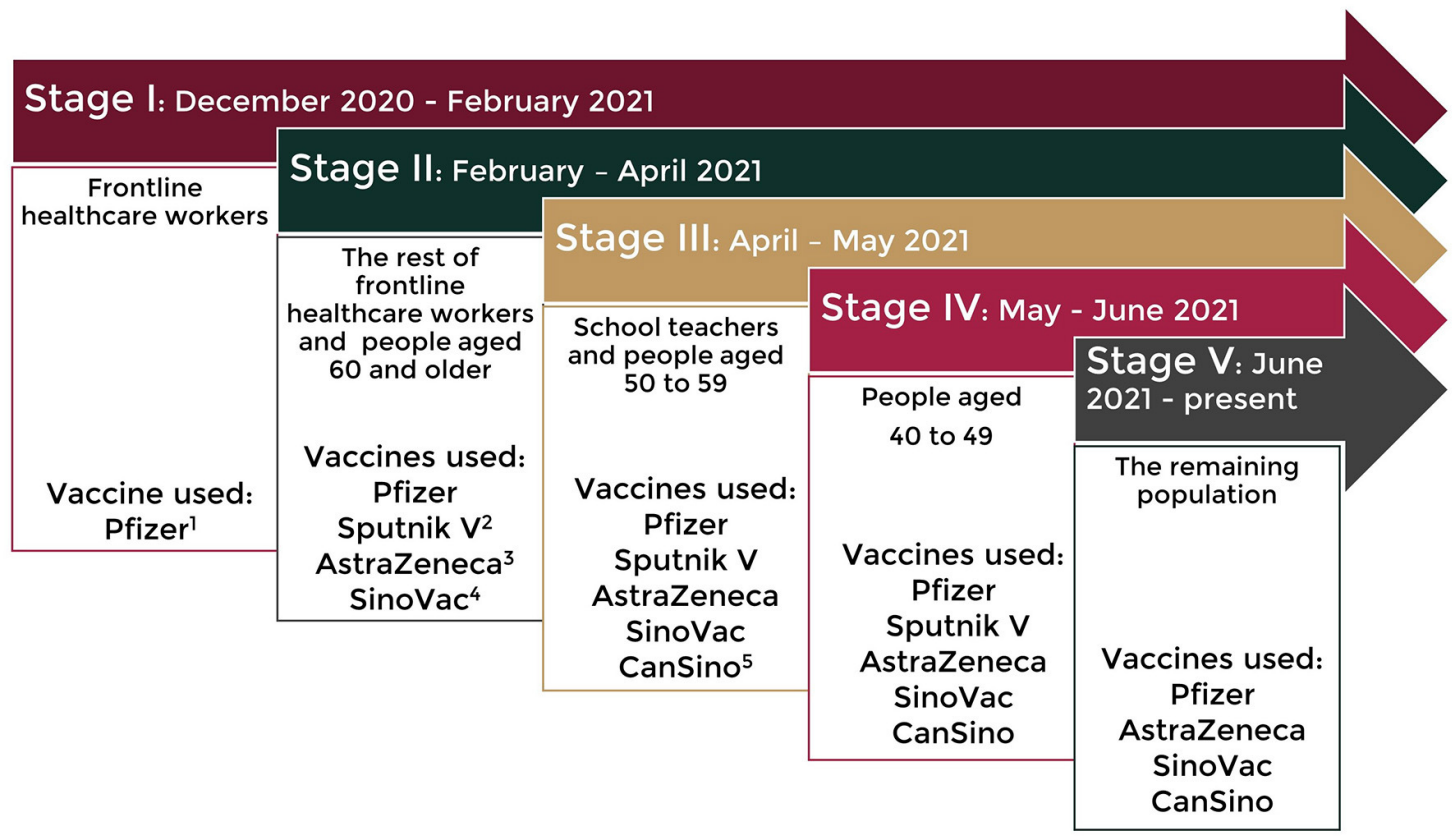
Vaccination strategy in Mexico. ^1^BNT162b2 (Pfizer-BioNTech), ^2^Gam-COVID-Vac (Gamaleya's Sputnik V), ^3^ChAdOx1 (AstraZeneca), ^4^CoronaVac (Sinovac Life Sciences), and ^5^Ad5-nCoV (CanSinoBIO).

By January 2020, the severe acute respiratory syndrome coronavirus 2 (SARS-CoV-2) infection that originated in Wuhan, China had already spread to Europe and by March 2020, it had already spread to the whole world (1–4). The number of people infected was rapidly reached stunning figures given its high transmissibility. The health services collapsed, and the loss of life was alarming in the absence of a specific treatment. Fortunately, Coronavirus Disease-19 (COVID-19) vaccines emerged in record time. Clinical trials began to show the efficacy and safety of vaccines, such as the mRNA-1273 vaccine (Moderna) (5), the BNT162b2 vaccine (Pfizer-BioNTech), and the Gam-COVID-Vac (Gamaleya's Sputnik V) (6). Therefore, regulatory agencies began authorizing their emergency use. Starting date and requirements for the public to receive the vaccine varied by region. In Mexico, vaccination started in December 2020 and the administration was in stages according to priority groups with vaccines varying in type upon availability (Figure 1) (7). Additionally, some Mexicans sought to receive a vaccine abroad, mainly the United States, where mRNA-1273 (Moderna), Ad26.CoV2.S (Johnson & Johnson/Janssen), and BNT162b2 (Pfizer-BioNTech) were available. Vaccine acceptability is key to the success of any vaccination program. Two nationally representative surveys on COVID-19 vaccination are available in Mexico. One, conducted from August to November 2020, identified 62.3% acceptance, 28.2% refusal, and 9.5% hesitancy (8). The second, conducted in November 2020, reported 82% acceptance. Although unlike the first, this study combined a doubtful answer with a definitive one (9). Reports on the progress of coverage of the vaccination strategy in the country showed 20% of the target population fully vaccinated by August 2021 (beginning of the data collection of the present study), and 59% by the end of January 2022 (10, 11).

Despite having proven their safety, COVID-19 vaccines are not exempt from adverse effects. Adverse reactions have been reported in more than 1 in 10 people in BNT162b2 (Pfizer-BioNTech), ChAdOx1 (AstraZeneca), and mRNA-1273 vaccine (Moderna) clinical trials. They usually happen shortly after the vaccination and are not associated with a serious or lasting illness (12–17). Moreover, some factors, such as young age, female sex, and prior COVID-19 infection, may increase the frequency of side effects (18–24). Pharmacovigilance activities are essential in any country. In Mexico, a web page is available for registration of vaccines' adverse effects directly by the public (25). However, there is no promotion of its use and much of the general population is unaware of its existence. Post-vaccination surveillance studies are needed worldwide because it is important to understand the frequency and regional variation of side effects and its potential impact on daily life, so that the public can successfully anticipate appropriate actions. A few studies examined the comparative side effects of COVID-19 vaccines (18, 21, 26) and the reactogenicity of more than three different types of vaccines has not been assessed on the same survey.

The objective of the present study was to compare the extension and severity of self-reported side effects of seven COVID-19 vaccines [BNT162b2 (Pfizer-BioNTech), ChAdOx1 (AstraZeneca), mRNA-1273 (Moderna), CoronaVac (Sinovac Life Sciences), Gam-COVID-Vac (Gamaleya's Sputnik V), Ad5-nCoV (CanSinoBIO), and Ad26.CoV2.S (Johnson & Johnson/Janssen)] in the Mexican population. A second objective was to evaluate the association of type of vaccine, sex, age, comorbidity, and history of allergies to the extent and severity of side effects.

## Materials and Methods

### Study Design

**Figure 2:**
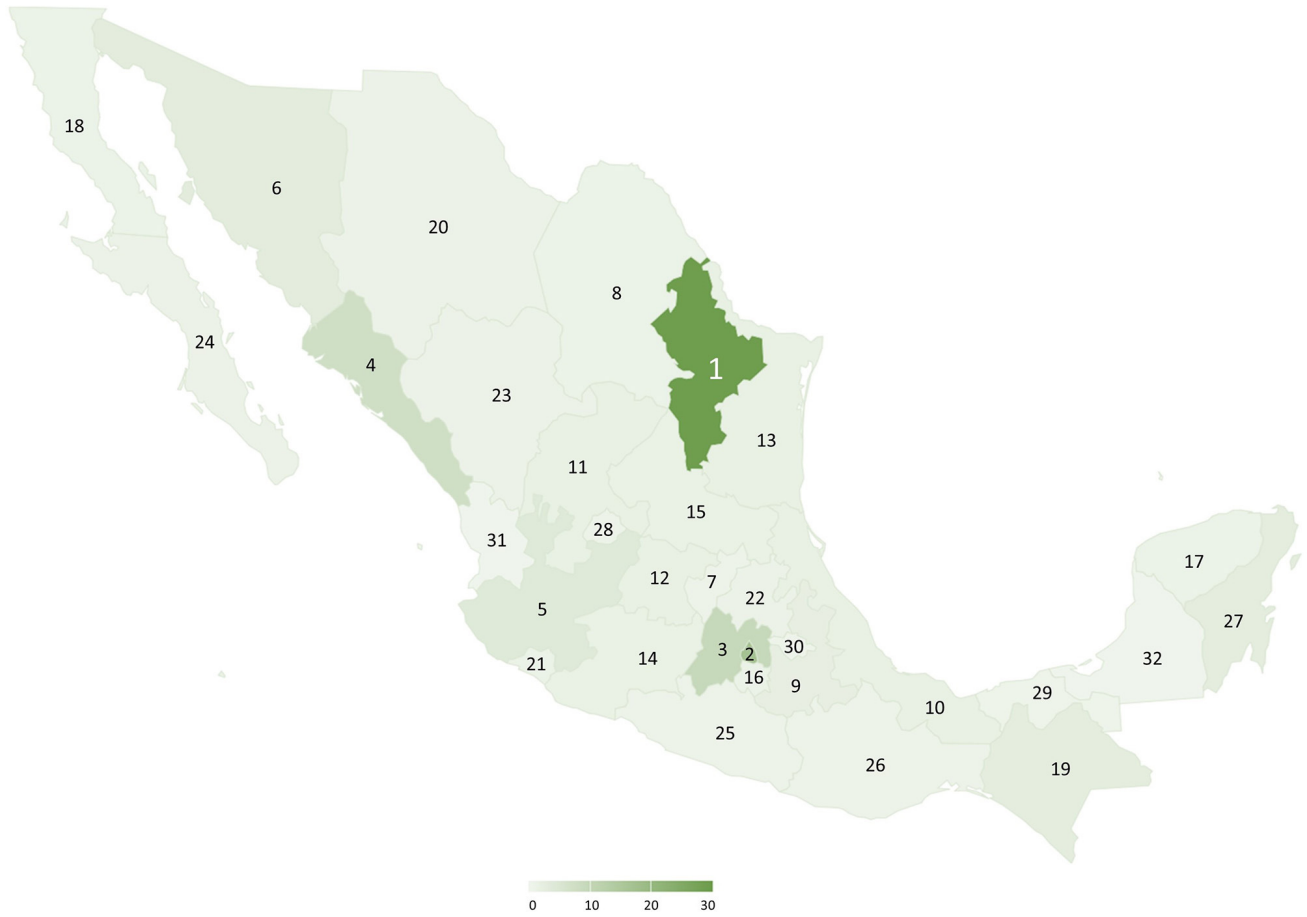
Geographical origin of participants and percentage of participation per state: 1. Nuevo León 29.2%, 2. Mexico City 16.7%, 3. State of Mexico 9.5%, 4. Sinaloa 7.4%, and 5–32. Rest of the country 0–5%. Study of Coronavirus Disease-2019 (COVID-19) vaccines' side effects in the Mexican population, August to September 2021.

This was a cross-sectional study carried out online between August 12 and September 3, 2021 in Mexico (two months after the vaccination was opened to all population groups). Nuevo Leon and Mexico City were the locations with more participants (Figure 2). The first inclusion criterion was to receive a COVID-19 vaccine and the second, being at least 18 years old. Those who did not sign the informed consent were excluded and there was no need to eliminate any registry due to lack of information on vaccine adverse effects. Non-random sampling based on the snowballing technique was used for recruiting potential participants who were invited through social media groups and word-of-mouth campaigns with no proportional quotas by sociodemographic variables or by type of vaccine. The survey link was distributed *via* multiple media platforms, such as Facebook and WhatsApp. Participants did not receive any kind of financial reward. Two sample sizes were estimated. One is based on the expected frequency of at least one local side effect between 48.9 and 85.8% reported in the literature (22, 27). The second is based on the expected frequency of at least one systemic side effect between 52.6 and 70.5% (22, 27). Both, with a margin of error of 3% and a CI of 95%. The minimum *n* required varied from 514 to 1,067 for estimates of local effects and between 879 and 1,064 for estimates of systemic effects. However, there were 4,024 participants. Such a sample size provided determinations with a precision of <2% and confidence level >95% given the frequencies obtained in the study of 67 and 65% for local and systemic side effects after the 1st dose (28). The protocol was approved by the Committees of Ethics and Health Research (R2021-1909-106). The study followed the Declaration of Helsinki for research on human subjects' guidelines (29). All the participants had to give their informed consent digitally before filling in the questionnaire. Participation was entirely voluntary, and withdrawal was allowed at any time without the need to justify the decision. There was no personal data collected that might enable the retrospective identification of the participant.

### Study Variables

#### Vaccine Information

Participants were asked about the type of vaccine received (multiple choice), vaccine combination (yes, no; if yes, type of combination), and the number of doses. Moreover, about the use of pre-vaccination medication to prevent symptoms (yes, no). For side effects evaluation, the participant chose all the symptoms that he/she had presented from a list made with short-term side effects reported in the literature (12–17). Then, the extension was classified as local, systemic, or both. To simplify the questionnaire as much as possible, the time of onset and duration for each side effect were not included. The side effects of dose 1 and dose 2 were questioned in separate sections. For severity evaluation, three questions were asked for first or single dose: need to take medicine, stop activities/miss work, or seek medical attention (went to a doctor, went to an emergency room, or was hospitalized). Then, an index was constructed based on negative and affirmative responses obtaining 3 categories: mild, moderate, and severe (Table 1). One question was used for the second dose: notice any difference in symptomatology between the first and second doses (felt better, felt the same, or felt worse).

**Table 1:** Severity categorization.

**Severity**	**Took medicine to relieve symptom**	**Suspended daily activity or missed work**	**Sought medical attention**
Mild	Yes	No	No
Moderate	Yes	Yes	No
Severe	Yes or No	Yes or No	Yes, regardless of the need to take medication or suspend activities/miss work

*Study on self-reported side effects of Coronavirus Disease-19 (COVID-19) vaccines in the Mexican population, August to September 2021*.

#### Comorbidity, Sociodemographic, and Other Characteristics

One question filtered the history of any comorbidity. Those who answered affirmatively were asked about a previous diagnosis of prediabetes, diabetes, hypertension, chronic renal failure, chronic obstructive lung disease, asthma, immune disease, cancer, cerebrovascular disease, others. Moreover, about the history of allergies and COVID-19 infection (had symptoms consistent with COVID-19 disease and was positive to PCR or rapid nasal swab antigen test). Sex, age, schooling, occupation, place of residence, smoking, pregnancy, and breastfeeding at the time of vaccination were identified too. Nutritional status was assessed using a validated body mass index-body size pictorial method that indicated low weight (shape 1), normal weight (shapes 2 and 3), overweight (shape 4), and obesity (shapes 5–10) (30).

### Study Procedures

The self-applied electronic survey was designed in Spanish with the software tool QuestionPro (Survey Analytics LLC, San Francisco, CA, USA) and Google Forms (Google, Mountain View, CA, USA). The survey took an average of 5 min to complete. It was divided into the following sections: privacy policy (statements about voluntary participation and confidentiality), vaccine data, post-vaccination side effects, COVID-19 prior infection, and comorbidity data; sociodemographic and other information of interest. The items were developed by the authors after a thorough review of the literature on side effects reported by the different vaccines. These were submitted to a panel of experts (an epidemiologist medical doctor and 2 full-time medical researchers) who reviewed the pertinence and relevance of the content. Moreover, they checked if writing was clear, concise, and unambiguous and verified the absence of technical language. The proposed questionnaire was pre-tested among colleagues, then it was tested in a pilot study with participants of sociodemographic characteristics similar to the target population. Some adjustments were made on section skipping according to filter questions. The final version is available in Supplementary Materials S1.

### Statistical Analysis

Frequencies were obtained for the categorical variables, as were means and SDs for the non-categorical variables. Point prevalence and 95% CIs were estimated. The association of sociodemographic, comorbidity, and history of allergies to extension and severity of side effects was evaluated through the chi-square tests. Then, stepwise multivariate logistic ordinal regression models were run to calculate adjusted odds ratio (OR) and 95% CI for each outcome of interest. One model included an extension of side effects as dependent variable (coded as absent, local, systemic, both) and type of vaccine, sex, age, comorbidity, allergies, and smoking as independent variables; use of pre-vaccination medication to prevent symptoms and history of confirmed COVID-19 infection were used as control variables. The model for analyzing severity included severity index (coded as absent, mild, moderate, and severe) or difference in symptomatology between first and second doses (coded as felt better, felt the same, or felt worse) as dependent variables. Outcomes were analyzed for dose 1 and dose 2, separately. Due to the small sample size, 4 records were removed from inferential statistical analysis, 1 BBIBP-CorV (Sinopharm, Beijing, China) registry, and 3 CureVac registries. Analyses were done using SPSS for Windows version 22.

## Results

The mean age was 38.9 ± 11.0 years, 3.6% were pregnant and 9.9% were breastfeeding when they got the vaccine. The female sex, the bachelor's school degree, and being employed or self-employed were characteristics that predominated in the study population. Some characteristics differed by type of vaccine. There were more participants between 30 and 39 years with ChAdOx1 (AstraZeneca), Gam-COVID-Vac (Gamaleya's Sputnik V), Ad5-nCoV (CanSinoBIO), and Ad26.CoV2.S (Johnson & Johnson/Janssen). Moreover, more participants with higher schooling and employed/self-employed with Ad5-nCoV (CanSinoBIO). Table 2 shows these and other detailed results. A very low percentage of combined vaccines (2%) and 67.4% had a complete scheme (two doses or one for single-shot vaccines). Less than 7% of participants used medication to prevent symptoms before the first or second vaccinations (6.9 and 6.5%, respectively, *p* = 0.549).

**Table 2:** Sociodemographic, comorbidity, and other characteristics.

		**Type of vaccine**							
	**Total**	**Pfizer^**a**^**	**AstraZeneca^**b**^**	**Moderna^**c**^**	**SinoVac^**d**^**	**Sputnik V^**e**^**	**Cansino^**f**^**	**J & J^**g**^**	**Chi-square *p*-value**
	***n* = 4,024**	***n* =1,579**	***n* =1,193**	***n* =52**	***n* =299**	***n* =202**	***n* =598**	***n* =97**	
Female	79.7%	79.4%	79.3%	76.9%	78.9%	84.2%	80.4%	79.4%	0.778
**Age group (years)**									
<29	18.8%	16.2%	19.5%	21.2%	27.2%	24.8%	17.1%	23.7%	
30–39	41.9%	36.7%	47.8%	28.8%	30.9%	49.0%	46.9%	50.5%	
40–49	21.2%	24.0%	20.1%	30.8%	16.8%	1.0%	25.6%	12.4%	
50 a 59	12.3%	16.3%	6.2%	11.5%	13.8%	19.8%	10.2%	13.4%	
≥60	5.8%	6.8%	6.4%	7.7%	11.4%	5.4%	0.2%	0.0%	0.0001
**Schooling**									
Middle school	3.0%	3.3%	4.0%	0.0%	5.0%	1.5%	0.3%	2.1%	
High school	11.3%	11.4%	14.9%	3.8%	19.1%	9.4%	2.2%	6.2%	
Bachelor's degree	50.1%	48.7%	52.1%	53.8%	53.5%	54.0%	44.8%	61.9%	
Postgraduate	35.5%	36.6%	28.9%	42.3%	22.4%	35.1%	52.7%	29.9%	0.0001
**Occupation**									
Employed/self-employed	71.6%	75.0%	63.5%	75.0%	59.2%	57.9%	90.1%	66.0%	
Housewife	12.1%	11.0%	16.2%	17.3%	15.7%	14.4%	1.7%	23.7%	
Retired/unemployed	7.7%	7.7%	9.3%	1.9%	11.4%	13.4%	2.3%	1.0%	
Student	8.6%	6.2%	11.1%	5.8%	13.7%	14.4%	5.9%	9.3%	0.0001
Smoking	13.1%	13.5%	14.6%	15.4%	13.7%	8.9%	10.5%	8.2%	0.075
Comorbidity (any)	19.3%	22.9%	16.3%	15.4%	18.4%	18.3%	18.9%	7.2%	0.0001
Hypertension	7.4%	9.4%	6.4%	9.6%	7.0%	6.4%	5.7%	2.1%	0.006
Prediabetes or diabetes	4.8%	6.2%	4.1%	1.9%	4.0%	3.0%	4.2%	1.0%	0.025
Allergies	30.9%	30.8%	30.9%	32.7%	28.4%	23.3%	34.8%	27.8%	0.085
COVID-19 before 1st dose	17.4%	22.0%	15.6%	17.3%	17.4%	11.9%	12.4%	7.2%	0.0001
Overweight/obese	54.7%	56.3%	53.5%	51.9%	54.8%	44.1%	57.9%	46.4%	0.010

*Study on self-reported side effects of Coronavirus Disease-19 (COVID-19) vaccines in the Mexican population, August to September 2021 (n = 4,024)*.

a *BNT162b2 (Pfizer-BioNTech)*.

b *ChAdOx1 (AstraZeneca)*.

c *mRNA-1273 (Moderna)*.

d *CoronaVac (Sinovac Life Sciences)*.

e *Gam-COVID-Vac (Gamaleya's Sputnik V)*.

f *Ad5-nCoV (CanSinoBIO)*.

g *Ad26.CoV2.S (Johnson & Johnson/Janssen)*.

### Extension of Side Effects

**Figure 3:**
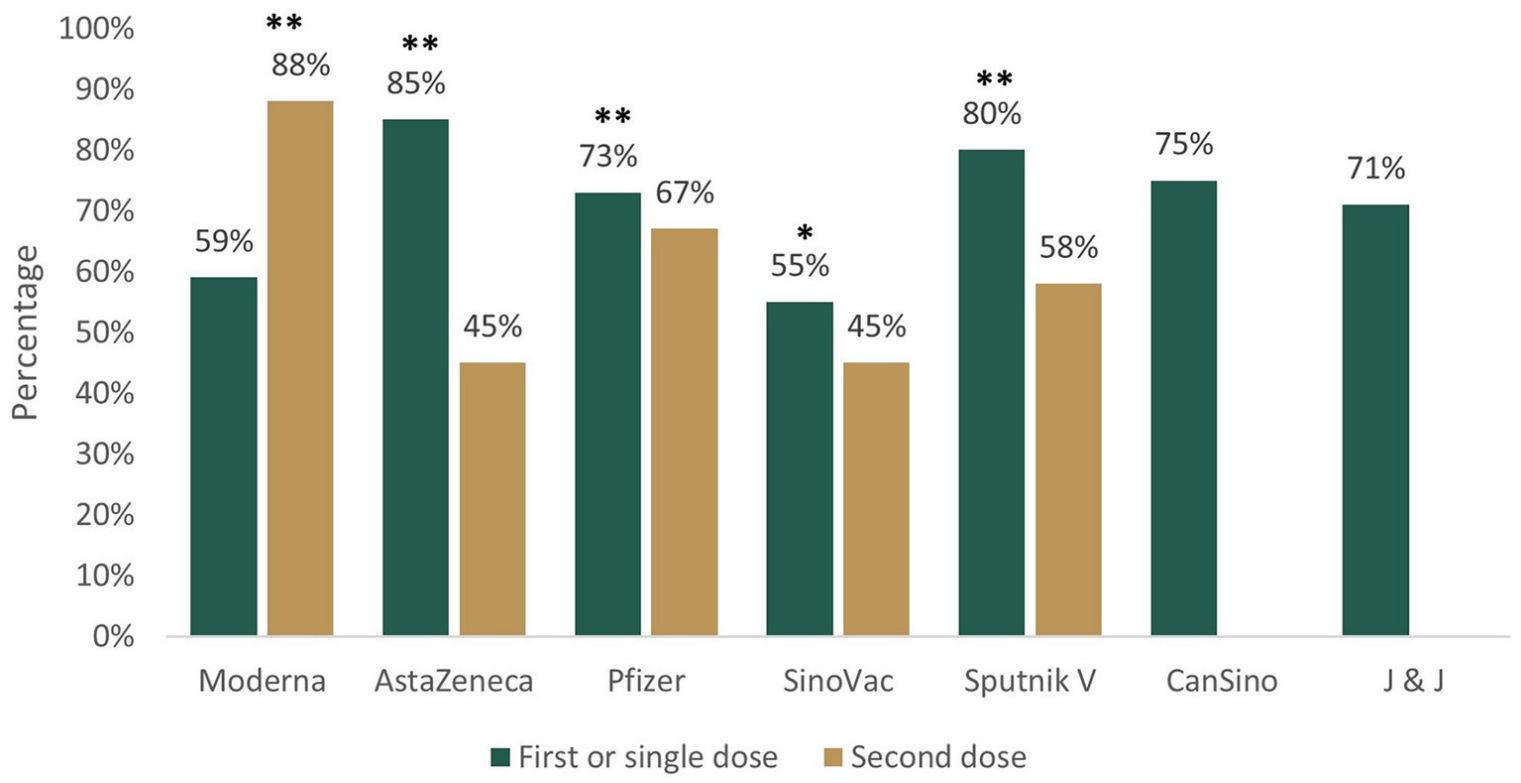
Prevalence of at least one side effect after first/single and second dose of Coronavirus Disease-2019 (COVID-19) vaccines in Mexican population, August to September 2021. ^*^p ≤ 0.05, ^**^p < 0.001 *Z*-tests for analyzing the difference between two proportions. Moderna (mRNA-1273), AstraZeneca (ChAdOx1), Pfizer (BNT162b2), Sinovac (CoronaVac), Sputnik V (Gam-COVID-Vac), CanSino (Ad5-nCoV), and J & J (Johnson & Johnson, Ad26.CoV2.S).

Prevalence of side effects varied by type of vaccines and number of doses. ChAdOx1 (AstraZeneca) was the vaccine with the highest prevalence of at least one side effect at dose 1 and mRNA-1273 (Moderna), at dose 2 (Figure 3). Stratification by local and systemic effects showed the prevalence of 67% (95% CI 65.1, 68.0) and 65% (95% CI 63.3, 66.2) after the first vaccination, respectively. The classification by extension showed that the combination of local and systemic effects was the most frequent category after the first dose, which exceeded that of the second dose. Arm/injection site pain was the most common local symptom and headache was the most common systemic symptom regardless of the number of doses (Table 3). Side effects categorized by organ system and the number of doses are provided in Supplementary Table S2. Side effects categorized by organ system and type of vaccine are provided in Supplementary Tables S3, S4.

**Table 3:** Extension of side effects by the number of doses.

	**First or single dose vaccination** **(*****n*** **= 4,024)**		**Second dose vaccination** **(*****n*** **= 2,050)**	
	***n***	**% (95% Confidence interval)**	***n***	**% (95% Confidence interval)**
Extension				
No symptoms	975	24.2 (22.9, 25.6)	778	38.0 (35.9, 40.1)
Local symptoms	443	11.0 (10.1, 12.0)	274	13.4 (12.0, 14.9)
Systemic symptoms	370	9.2 (8.3, 10.1)	203	9.9 (8.7, 11.3)
Both, local and systemic symptoms	2,236	55.6 (54.0, 57.1)	795	38.8 (36.7, 40.9)
Type of local side effect^a^				
Arm /injection site pain	2,650	65.9 (64.4, 67.3)	1,061	51.8 (49.6, 53.9)
Injection site swelling	334	8.3 (7.5, 9.2)	103	5.0 (4.2, 6.1)
Injection site itching	210	5.2 (4.6, 5.9)	54	2.6 (2.0, 3.4)
Injection site redness	168	4.2 (3.6, 4.8)	50	2.4 (1.9, 3.2)
Type of systemic side effect^a^				
Headache	1,537	38.2 (36.7, 39.7)	541	26.4 (24.5, 28.3)
Muscle pain	1,293	32.1 (30.7, 33.6)	370	18.0 (16.4, 19.8)
Lack of energy	1,115	27.7 (26.3, 29.1)	312	15.2 (13.7, 16.8)
Fatigue or tiredness	1,107	27.5 (26.2, 28.9)	477	23.3 (21.5, 25.1)
Fever	926	23.0 (21.7, 24.3)	236	11.5 (10.2, 13.0)
Desire to sleep	912	22.7 (21.4, 24.0)	300	14.6 (13.2, 16.2)
Chills	817	20.3 (19.1, 21.6)	214	10.4 (9.2, 11.8)
Malaise	760	18.9 (17.7, 20.1)	259	12.6 (11.3, 14.1)
Bone or joint pain	713	17.7 (16.6, 18.9)	222	10.8 (9.6, 12.2)
Nausea	274	6.8 (6.1, 7.6)	69	3.4 (2.7, 4.2)
Dizziness and giddiness	265	6.6 (5.9, 7.4)	76	3.7 (3.0, 4.6)
Hot flashes	255	6.3 (5.6, 7.1)	60	2.9 (2.3, 3.7)
Eye movement pain	253	6.3 (5.6, 7.1)	55	2.7 (2.1, 3.5)
Sweating	236	5.9 (5.2, 6.6)	55	2.7 (2.1, 3.5)
Stuffy nose	197	4.9 (4.3, 5.6)	61	3 (2.3, 3.8)
Sore throat	191	4.7 (4.1, 5.4)	72	3.5 (2.8, 4.4)
Diarrhea	177	4.4 (3.8, 5.1)	71	3.5 (2.8, 4.3)
Chest pain	174	4.3 (3.7, 5.0)	45	2.2 (1.6, 2.9)
A faster or lower heartbeat	166	4.1 (3.6, 4.8)	41	2.0 (1.5, 2.7)
Irritated eyes	130	3.2 (2.7, 3.8)	31	1.5 (1.1, 2.1)
Running nose	124	3.1 (2.6, 3.7)	54	2.6 (2, 3.4)
Difficulty breathing (dyspnea)	107	2.7 (2.2, 3.2)	25	1.2 (0.8, 1.8)
Abdominal pain	96	2.4 (2.0, 2.9)	25	1.2 (0.8, 1.8)
Lymph nodes tenderness	91	2.3 (1.8, 2.8)	38	1.9 (1.4, 2.5)
Cough	90	2.2 (1.8, 2.7)	34	1.7 (1.2, 2.3)
Rise in blood pressure	87	2.2 (1.8, 2.7)	15	0.7 (0.4, 1.2)
Vomiting	57	1.4 (1.1, 1.8)	20	1.0 (0.6, 1.5)
Skin rash, hives, irritable skin	50	1.2 (0.9, 1.6)	11	0.5 (0.3, 1.0)
Loss of blood pressure	44	1.1 (0.8, 1.5)	7	0.3 (0.2, 0.7)
Other	92	2.3 (1.9, 2.8)	23	1.1 (0.7, 1.7)

*Study on self-reported side effects of Coronavirus Disease-19 (COVID-19) vaccines in the Mexican population, August to September 2021*.

a *Ordered from highest to lowest frequency after first dose*.

### Severity of Side Effects

After dose 1, 62.6% needed to take medicine for relieving symptoms, 31.3% suspended daily activities or missed work, and 5.3% sought medical attention. The severity index showed 28% had mild, 21% moderate, and 4% severe effects; the rest did not experience symptoms, did not take medication, stopped activities, or sought medical attention (47%). The severity of side effects differed by type of vaccine. At dose 1, there were more respondents suspending activities/missing work and taking medicine for relieving symptoms with ChAdOx1 (AstraZeneca) and more seeking medical attention with Ad5-nCoV (CanSinoBIO) (Table 4).

**Table 4:** The severity of side effects by the number of dose and type of vaccine.

	**Type of vaccine**							
	**Pfizer^**a**^**	**AstraZeneca^**b**^**	**Moderna^**c**^**	**SinoVac^**d**^**	**Sputnik V^**e**^**	**Cansino^**f**^**	**J & J^**g**^**	**Chi-square** ***p*-value**
Dose 1								
Suspended daily activities/missed work	164 (14.2%)	498 (49.2%)	5 (13.9%)	39 (23.8%)	63 (39.1%)	168 (37.7%)	16 (23.2%)	0.0001
Sought medical attention	42 (3.6%)	67 (6.6%)	1 (2.8%)	8 (4.9%)	5 (3.1%)	37 (8.3%)	(1) 1.4%	0.001
Took medicine	598 (51.7%)	804 (79.4%)	27 (75%)	69 (42.1%)	105 (65.2%)	252 (56.4%)	52 (75.4%)	0.0001
Severity index								
None	935 (59.2%)	312 (26.2%)	24 (46.2%)	212 (70.9%)	75 (37.1%)	289 (48.3%)	43 (44.3%)	
Mild	462 (29.3%)	369 (30.9%)	22 (42.3%)	46 (15.4%)	60 (29.7%)	128 (21.4%)	37 (38.1%)	
Moderate or severe	182 (11.5%)	512 (42.9%)	6 (11.5%)	41 (13.7%)	67 (33.2%)	181 (30.3%)	17 (17.5%)	0.0001
Dose 2								
Felt better	434 (31.5%)	190 (56.2%)	5 (10%)	41 (23.2%)	31 (43.7%)	–	–	
Felt the same	561 (40.7%)	107 (31.7%)	18 (36%)	105 (59.3%)	25 (35.2%)	–	–	
Felt worse	383 (27.8%)	41 (12.1%)	27 (54%)	31 (17.5%)	15 (21.1%)	–	–	0.0001

*Study on self-reported side effects of Coronavirus Disease-19 (COVID-19) vaccines in the Mexican population, August to September 2021*.

a *BNT162b2 (Pfizer-BioNTech)*.

b *ChAdOx1 (AstraZeneca)*.

c *mRNA-1273 (Moderna)*.

d *CoronaVac (Sinovac Life Sciences)*.

e *Gam-COVID-Vac (Gamaleya's Sputnik V)*.

f *Ad5-nCoV (CanSinoBIO)*.

g *Ad26.CoV2.S (Johnson & Johnson/Janssen)*.

### Factors Associated to Extension of Side Effects

At dose 1, participants with ChAdOx1 (AstraZeneca) and Gam-COVID-Vac (Gamaleya's Sputnik V) were more likely to have local and systemic side effects than BNT162b2 (Pfizer-BioNTech). In contrast, participants with CoronaVac (Sinovac Life Sciences) were less likely to have such an outcome. At dose 2, mRNA-1273 (Moderna) increased the odds of greater side effects extension, while ChAdOx1 (AstraZeneca) and CoronaVac (Sinovac Life Sciences) decreased them. Factors, such as female sex, age under 50, and history of allergies, increased the possibilities of greater extension at doses 1 and 2, regardless of comorbidity, smoking, pre-vaccination medication to prevent symptoms, and history of confirmed COVID-19 infection (Table 5).

**Table 5:** Multivariate ordinal regression analyses of factors associated to an extension of side effects in Mexican population, August to September 2021.

	**Extension**					
	**Absent**	**Local**	**Systemic**	**Local and systemic**	**Adjusted odds ratios**^**a**^ **(95% CI)**	**Wald Chi-square** ***p*-value**
**First dose**						
Type of vaccine						
Pfizer^1^	43.4%	71.1%	20.0%	34.4%	1.00	
AstraZeneca^2^	18.6%	8.6%	36.5%	37.6%	2.53 (2.16, 2.96)	0.00001
Cansino^3^	15.5%	7.4%	26.5%	14.2%	1.18 (0.99, 1.42)	0.072
SinoVac^4^	13.8%	8.6%	7.6%	4.4%	0.52 (0.41, 0.66)	0.00001
Sputnik V^5^	4.2%	1.4%	4.9%	6.1%	2.41 (1.76, 3.29)	0.00001
J & J^6^	2.9%	0.2%	4.3%	2.3%	1.27 (0.85, 1.91)	0.246
Moderna^7^	1.6%	2.7%	0.3%	1.0%	0.82 (0.49, 1.38)	0.465
Female sex	73.5%	78.6%	77.8%	82.9%	1.45 (1.25, 1.68)	0.00001
Age < 50 years	70.8%	77.9%	78.6%	88.1%	2.29 (1.95, 2.69)	0.00001
Comorbidity (any)	19.7%	21.0%	16.5%	19.3%	1.19 (1.01, 1.41)	0.033
Allergies	23.0%	27.8%	27.6%	35.5%	1.58 (1.37, 1.81)	0.00001
Smoking	13.8%	12.4%	11.1%	13.2%	1.04 (0.87, 1.26)	0.645
**Second dose**						
Type of vaccine						
Pfizer^1^	58.5%	76.6%	68.2%	75.4%	1.00	
AstraZeneca^2^	24.1%	11.7%	20.4%	10.3%	0.46 (0.36, 0.58)	0.0001
SinoVac^4^	12.6%	8.8%	6.5%	5.6%	0.47 (0.35, 0.63)	0.0001
Sputnik V^5^	3.9%	1.5%	2.0%	4.3%	1.29 (0.80, 2.08)	0.294
Moderna^7^	0.8%	1.5%	3.0%	4.4%	2.88 (1.59, 5.21)	0.0001
Female sex	76.9%	79.9%	79.8%	83.4%	1.27 (1.03, 1.57)	0.025
Age <50 years	57.7%	73.7%	63.1%	79.6%	2.03 (1.68, 2.45)	0.0001
Comorbidity (any)	25.2%	27.0%	26.1%	22.1%	1.04 (0.85, 1.26)	0.349
Allergies	26.0%	29.9%	35.0%	33.1%	1.2 (1.00, 1.44)	0.728
Smoking	14.1%	11.3%	10.3%	14.5%	1.13 (0.88, 1.44)	0.046

a *Adjusted by the preventive use of medication to prevent symptoms before vaccination and history of confirmed COVID-19 infection*.

1 *BNT162b2 (Pfizer-BioNTech)*.

2 *ChAdOx1 (AstraZeneca)*.

3 *mRNA-1273 (Moderna)*.

4 *CoronaVac (Sinovac Life Sciences)*.

5 *Gam-COVID-Vac (Gamaleya's Sputnik V)*.

6 *Ad5-nCoV (CanSinoBIO)*.

7 *Ad26.CoV2.S (Johnson & Johnson/Janssen)*.

### Factors Associated to Severity of Side Effects

At dose 1, four vaccines were associated to greater severity: ChAdOx1 (AstraZeneca), Ad5-nCoV (CanSinoBIO), Gam-COVID-Vac (Gamaleya's Sputnik V), and Ad26.CoV2.S (Johnson & Johnson/Janssen). Female sex, age <50 years, comorbidity, and allergies were also associated factors, independent of smoking, pre-vaccination medication to prevent symptoms, and history of confirmed COVID-19 infection (Table 6). At dose 2, mRNA-1273 (Moderna) tripled the possibilities of feeling worse compared to the first dose (95% CI 1.82, 5.43; *p* < 0.0001). In contrast, ChAdOx1 (AstraZeneca) (adjusted OR 0.34, 95% CI 0.26, 0.42; *p* < 0.0001) and Gam-COVID-Vac (Gamaleya's Sputnik V) (adjusted OR 0.59, 95% CI 0.37, 0.94; *p* = 0.025) reduced them.

**Table 6:** Multivariate ordinal regression analyses of factors associated to the severity of side effects in Mexican population, August to September 2021.

	**Severity**					
	**Absent**	**Mild**	**Moderate**	**Severe**	**Adjusted odds ratios**^**a**^ **(95% CI)**	**Wald Chi-square** ***p*-value**
**First dose**						
Type of vaccine						
Pfizer^1^	49.5%	41.1%	16.6%	26.1%	1.00	
AstraZeneca^2^	16.5%	32.8%	52.7%	41.6%	4.32 (3.73, 5.0)	0.0001
Cansino^3^	15.3%	11.4%	17.0%	23.0%	1.96 (1.63, 2.36)	0.0001
SinoVac^4^	11.2%	4.1%	3.9%	5.0%	0.71 (0.54, 0.93)	0.013
Sputnik V^5^	4.0%	5.3%	7.3%	3.1%	3.00 (2.28, 3.94)	0.0001
J & J^6^	2.3%	3.3%	1.9%	0.6%	1.68 (1.15, 2.46)	0.008
Moderna^7^	1.3%	2.0%	0.6%	0.6%	1.43 (0.86, 2.38)	0.163
Female sex	75.4%	83.3%	83.3%	85.7%	1.52 (1.30, 1.78)	0.0001
Age < 50 years	77.2%	83.9%	88.9%	85.7%	1.58 (1.30, 1.87)	0.0001
Comorbidity (any)	18.8%	19.9%	18.9%	23.6%	1.22 (1.05, 1.43)	0.011
Allergies	25.7%	33.6%	37.3%	39.1%	1.49 (1.31, 1.70)	0.0001
Smoking	12.5%	13.5%	13.6%	13.7%	1.11 (0.93, 1.33)	0.233

a *Adjusted by the preventive use of medication to prevent symptoms before vaccination and history of confirmed COVID-19 infection*.

1 *BNT162b2 (Pfizer-BioNTech)*.

2 *ChAdOx1 (AstraZeneca)*.

3 *mRNA-1273 (Moderna)*.

4 *CoronaVac (Sinovac Life Sciences)*.

5 *Gam-COVID-Vac (Gamaleya's Sputnik V)*.

6 *Ad5-nCoV (CanSinoBIO)*.

7 *Ad26.CoV2.S (Johnson & Johnson/Janssen)*.

## Discussion

In the present study, we analyzed and explored associated factors to adverse reactions after COVID-19 vaccination from different laboratories and schemes (2 single-dose and 5 double-dose vaccines) in the Mexican population characterized by being in their 30s, being a woman, and with high schooling.

Prevalence of at least one side effect after the first dose varied between vaccines. CoronaVac (Sinovac Life Sciences) registered the lowest frequency (55%), which was higher than phases 2 and 3 clinical trials reports with values between 19 and 33% (31, 32). The next vaccine with less adverse effects was mRNA-1273 (Moderna) (69%), lower than 84% documented in a phase 3 clinical trial (33). Three vaccines showed prevalence around 70%, BNT162b2 (Pfizer-BioNTech; 73%), Ad5-nCoV (CanSinoBIO; 75%), and Ad26.CoV2.S (Johnson & Johnson/Janssen; 71%). The BNT162b2 (Pfizer-BioNTech) statistics of side effects are contrasting. An ongoing multinational placebo-controlled clinical trial reported 27% (34), but surveys with self-reported symptoms showed figures between 80 and 92% (21, 22, 35). Ad5-nCoV (CanSinoBIO) and Ad26.CoV2.S (Johnson & Johnson/Janssen) frequencies were close to the ones reported in phase 2 clinical trials. Zhu et al. (36) found 72–74% with Ad5-nCoV (CanSinoBIO) and Sadoff et al. (37) identified 62–63% with low Ad26.CoV2.S (Johnson & Johnson/Janssen) dose and 78–82% with high dose. The vaccines with the highest frequency of side effects were ChAdOx1 (AstraZeneca) and Gam-COVID-Vac (Gamaleya's Sputnik V) (85 and 80%, respectively). Both were within the range of self-report surveys. The former has shown prevalence ranging from 51 to 96% (21, 27, 35, 38, 39) and the second one, prevalence ranging from 71 to 82% (40, 41).

### First vs. Second Doses

We found a stronger reaction to the first than the second dose. ChAdOx1 (AstraZeneca) presented the highest difference with +40%, Gam-COVID-Vac (Gamaleya's Sputnik V) with +22%, and CoronaVac (Sinovac Life Sciences) with +10%. The stronger reaction to ChAdOx1 (AstraZeneca) first dose was in line with other reports (14, 42). Jarynowski et al. (20) also showed a higher average of adverse effects with the first than the second dose Gam-COVID-Vac (Gamaleya's Sputnik V) (2.2 ± 1.8 vs. 1.9 ± 1.7). They attributed such a result to the vaccine vector used in dose 2, which is different from dose 1. BNT162b2 (Pfizer-BioNTech) registered an overall difference of +6 indicating higher side effects with dose 1, unlike other studies that had reported more effects with the second dose (15, 18, 19). The discrepancy may be due to differences in age, sex, comorbidities, and history of COVID-19 infection. In addition, to geographic region and immune response variations. A systematic review and meta-analysis conducted by Choe et al. (43) showed that the geographic region was an important source of variation in the immune response to pneumococcal conjugate vaccines. Further research is needed to identify the reasons for an observed result contrary to what was expected. mRNA-1273 (Moderna) was the only vaccine with higher side effects at dose 2, which was in accordance with what the Centers for Disease Control and Prevention (CDC) reports. That is mRNA-1273 (Moderna) tends to present higher symptomatology the second time (16).

### Extension of Side Effects

Unlike other studies, this one distinguished the frequency of local and systemic symptoms in a single or combined presentation. At dose 1, very few had local or systemic symptoms in solitary instead they experienced both, which was considered of greater extension. ChAdOx1 (AstraZeneca) and Gam-COVID-Vac (Gamaleya's Sputnik V) were associated to greater extension while CoronaVac (Sinovac Life Sciences) to lesser extension, independent of sex, age, and other potential confounders. At dose 2, the absence of symptoms equaled the category of a combination of local and systemic symptoms and mRNA-1273 (Moderna) was the only vaccine associated with the greater extension. Regarding local symptoms, we identified important differences with the literature. For example, BNT162b2 (Pfizer-BioNTech) and ChAdOx1 (AstraZeneca) each registered 50% of participants with pain at the injection site in dose 1, which was less than that reported before between 68 and 81.2% (18, 21). At dose 2, the rates were 34.5 and 18%, respectively. Moreover, lower than in other studies >70% (18, 44). The rates of fatigue and muscle pain secondary to CoronaVac (Sinovac Life Sciences) in dose 1 were lower than those identified by Djanas et al. (45) (13% vs. 35.8% and 39.6%, respectively). Moreover, in dose 2, BNT162b2 (Pfizer-BioNTech) registered half the frequency reported elsewhere (18, 44). These differences remind the importance of estimating the prevalence of adverse effects in different populations. On the other hand, ChAdOx1 (AstraZeneca) registered higher systemic side effects than BNT162b2 (Pfizer-BioNTech) in dose 1, which was not surprising as this has been pointed out by other authors (18, 21).

### The Severity of Side Effects

The severity of side effects varied by the type of vaccine and the number of doses. Four vaccines increased the possibilities of greater severity at the first dose, ChAdOx1 (AstraZeneca), Ad5-nCoV (CanSinoBIO), Gam-COVID-Vac (Gamaleya's Sputnik V), and Ad26.CoV2.S (Johnson & Johnson/Janssen), as compared to BNT162b2 (Pfizer-BioNTech), independent of sex, age, and other potential confounders. There were more respondents suspending activities/missing work and taking medicine for relieving symptoms with ChAdOx1 (AstraZeneca). Kim et al. (18) also reported ChAdOx1 (AstraZeneca) with a higher impact on work productivity (work performance was impaired, took vacation or holiday, missed work). We found more participants seeking medical attention with Ad5-nCoV (CanSinoBIO) followed by ChAdOx1 (AstraZeneca). Kim et al. (18) also identified the latter vaccine with a higher frequency of subsequent need for going to a doctor, an emergency room, or being hospitalized.

### Associated Factors

Age, sex, and allergies were associated with greater extension and severity of side effects regardless of the type of vaccine and number of doses. Age <50 years presented the greatest risk, which doubled the chances of having local combined with systemic symptoms. Studies from different regions and different vaccines have documented respondents under 50 years with a higher risk of side effects (18, 20, 22, 27). It has been attributed to a decline in the function of the immune system with age. Women have a higher risk of adverse effects too (18–22). The disparity has been explained by differences in the immune response between men and women (46, 47). We also identified a history of allergies associated to greater extension and severity of side effects. Jahan et al. (27) found 45.1% of their participants had a history of allergic reactions to various allergens, which were associated with sneezing, coughing, itching, swelling, runny nose, and shortness of breath following the first application of ChAdOx1 (AstraZeneca). It appeared that the relative incidence of allergic reactions following administration of BNT162b2 (Pfizer-BioNTech) and mRNA-1273 (Moderna) vaccines had been higher for recipients with a prior history of allergies and/or anaphylaxis, respectively (48).

### Limitations

Differences in the use of certain vaccines were consistent with the vaccination program in the country, which in turn was a function of vaccines availability. The distribution by age, sex, and schooling was not as heterogeneous as would have been desired; and there was a bias toward young age, female sex, and higher schooling. Some reasons may help to explain it. In Mexico, there are more women than men (51 vs. 49%) and people between 15 and 49 years make up more than half of the population according to the 2020 population census (49). Women tend to participate more in health surveys. The higher presence of young adults reflects their greater familiarity and use of social media compared to older adults. Other authors have also reported more women and young respondents (18–21); and a higher frequency of university or post-university participants in online surveys (19, 27). The combination of higher education and a job with the Ad5-nCoV (CanSinoBIO) can be explained by the fact that this vaccine was applied mainly to schoolteachers and university professors. Additionally, diabetes and hypertension were half the frequency reported in the 2021 National Health and Nutrition Survey (50), probably secondary to the low presence of older adults. These were the ones with the least participation, which agrees with the percentage distribution of population 60 and over that according to the census ranks second least in the country, after that of 50–59 years (12 and 10%, respectively) (49). Future research requires the inclusion of a greater number of men, older adults, and less educated individuals. The study relied on self-report and symptoms were not verified. Some respondents may have incorrectly blamed the vaccine for the experienced side effect. Moreover, those who experienced side effects might have been more interested in participating than those who did not; and rates might be overestimated. Results might have been affected by memory bias. The use of mobile devices for reporting side effects in real-time might produce more accurate rates. Finally, the study focused on the short-term side effects; more research is needed for long-term effects.

## Conclusions

Prevalence and degree of adverse reactions differed by the number of doses and type of vaccine. At dose 1, ChAdOx1 (AstraZeneca) was the vaccine with the highest rate of at least one side effect followed by Gam-COVID-Vac (Gamaleya's Sputnik V). Both were associated with greater extension and severity of side effects. ChAdOx1 (AstraZeneca) was the vaccine in which more participants were required to suspend everyday duties or had to miss work. Young age (<50 years), female sex, comorbidity, and history of allergies were associated with greater extension and severity of side effects after the first vaccination, regardless of the type of vaccine and potential confounders. At dose 2, mRNA-1273 (Moderna) was the vaccine with the highest rate of side effects and the only vaccine associated with greater extension and severity of symptoms. Female sex and age under 50 increased the odds of greater extension after the second vaccination. Therefore, after receiving the COVID-19 vaccination, recipients should be advised about potential vaccine symptoms according to the number of doses, the type of vaccine, sex, age, and history of allergies. An informed public will know what to expect, what to do, when and where to seek additional guidance if necessary. Furthermore, measures for preventing or eliminating the unwanted effect might be planned. Continuous studies are necessary to acknowledge more about the post-vaccine symptoms in different populations.

## Data Availability Statement

The raw data supporting the conclusions of this article will be made available by the authors, without undue reservation.

## Ethics Statement

The studies involving human participants were reviewed and approved by Local Committee of Health Research No. 1909, Mexican Social Security Institute. The patients/participants provided their written informed consent to participate in this study.

## Institutional Review Board Statement

The study was conducted according to the guidelines of the Declaration of Helsinki and approved by the Institutional Review Board (or Ethics Committee) of the Mexican Social Security Institute (protocol 2021-1909-106, August 9, 2021).

## Author Contributions

MC and AS conceptualized the study and contributed to methodology, software, and writing—original draft preparation. BT and AS validated the data. MC, BT, and AS contributed to formal analysis. MC investigated the study. MC, AS, and JG contributed to data curation. MB, GN, JG, and BT contributed to writing, reviewing, and editing. AS and MB contributed to supervision. All authors have read and agreed to the published version of the manuscript.

## Conflict of Interest

The authors declare that the research was conducted in the absence of any commercial or financial relationships that could be construed as a potential conflict of interest.

## Publisher's Note

All claims expressed in this article are solely those of the authors and do not necessarily represent those of their affiliated organizations, or those of the publisher, the editors and the reviewers. Any product that may be evaluated in this article, or claim that may be made by its manufacturer, is not guaranteed or endorsed by the publisher.
