# Supplementary material for: Extension and Severity of Self-Reported Side Effects of Seven COVID-19 Vaccines in Mexican Population
Source: Front Public Health. 2022 Mar 14;10:834744. doi: 10.3389/fpubh.2022.834744 (PMC8964147; doi:10.3389/fpubh.2022.834744)
Supplement: Supplementary file 1 [file Table_1.DOCX]

S1. Questionnaire of the study on side effects of COVID-19 vaccines in the Mexican population

El presente cuestionario es anónimo y formará parte de una investigación que se llevará a cabo en el Centro de Investigación Biomédica del Noreste del Instituto Mexicano del Seguro Social. Este cuestionario tiene un tiempo de llenado de máximo 10 minutos y puede regresar a editar las respuestas pulsando el botón con la flecha que apunta hacia la izquierda, en caso de dudas puede contactar a la Dra. María Elena Camacho Moll cuyo correo electrónico es maria.camachomo@imss.gob.mx.El presente estudio no implica daño alguno y usted tiene la libertad de suspender el llenado de la encuesta si así lo considera. No se ocasionaría ningún daño en caso de que no decida participar, se garantiza la privacidad. Los datos generados serán usados con fines académicos y siguiendo la Ley General de Protección de Datos Personales en Posesión de Sujetos Obligados 17-01-2017 y en concordancia con el Reglamento de la Ley General de Salud en Materia de Investigación para la Salud DOF 2014.

Acepto la política de privacidad de la Ley General de Protección de Datos Personales en Posesión de Sujetos Obligados y las consideraciones éticas de la Ley General de Salud en Materia de Investigación para la Salud y La declaración de Helsinki de la Asociación Médica Mundial

1. Acepto

RESPONSABLE: Nombre: Dra. María Elena Camacho Moll / Departamento de Biología Molecular del Centro de Investigación Biomédica del Noreste Dirección electrónica: [maria.camachomo@imss.gob.mx](mailto:maria.camachomo@imss.gob.mx)

OBJETIVO:Recopilar datos sobre los efectos adversos de la vacunación contra COVID-19 en una población mexicana.

LEGITIMACIÓN:Se necesita otorgar el consentimiento para participar.

DERECHOS:En caso de querer suprimir/limitar datos, favor de enviar un correo electrónico a maria.camachomo@imss.gob.mx

Información sobre la vacuna

1. ¿Ya recibió la vacuna contra el COVID-19?

1. No
2. Sí

2. ¿Cuál vacuna le aplicaron?

1. Pfizer/BioNtech
2. AstraZeneca
3. Moderna
4. SinoVac
5. Sputnik V
6. CanSino
7. Johnson &amp; Johnson
8. No sabe/No se acuerda
9. Otra, ¿Cuál? __________

3. Algunas personas combinaron vacunas de diferentes laboratorios, ¿Es este su caso?

1. No
2. Sí

4. ¿Cuáles se aplicó de diferente laboratorio?, es decir, ¿Cuál con cuál? poniendo primero la vacuna de la primera dosis

|  |
| --- |

Efectos secundarios post-vacunación

5. ANTES DE LA VACUNACIÓN, ¿Consumió algún medicamento PARA PREVENIR los síntomas de la primer o única dosis de vacuna?

1. No
2. Sí, ¿Cuál? __________

6. ¿Presentó alguna molestia o síntoma después de la primera o única dosis de vacuna?

1. No
2. Sí

7. Después de la primera o única dosis de la vacuna, ¿presentó alguno de las siguientes molestias o síntomas? Seleccione todos los que apliquen

1. Comezón en el sitio de aplicación
2. Enrojecimiento el sitio de aplicación
3. Hinchazón en el sitio de aplicación
4. Dolor en el sitio de aplicación
5. Dolor en el brazo
6. Dolor de cabeza
7. Dolor de garganta
8. Dolor de músculos
9. Dolor de huesos
10. Dolor en el pecho
11. Dolor de abdomen
12. Dolor al mover los ojos
13. Elevación de la presión arterial
14. Baja presión
15. Bochornos
16. Congestión nasal
17. Debilidad
18. Desmayo o pérdida del conocimiento
19. Diarrea
20. Escalofríos
21. Escurrimiento nasal
22. Falta de aire
23. Fatiga o cansancio
24. Exceso de sueño
25. Fiebre
26. Inflamación de cara o cuello
27. Inflamación de ganglios
28. Irritación de ojos
29. Malestar general
30. Manchas en la piel
31. Mareo
32. Náuseas
33. Palpitaciones o taquicardia
34. Ronchas
35. Sangrado
36. Sudores
37. Tos
38. Vómito
39. Otro, ¿Cuál? __________

8. DESPUES DE LA VACUNACIÓN ¿Consumió algún medicamento o remedio para aliviar los síntomas?

1. No
2. Sí, ¿Cuál? __________

9. ¿ Le fue necesario buscar atención médica para aliviar los síntomas?

1. No
2. Sí, acudí con un médico
3. sí, acudí a un servicio de urgencias
4. sí, me hospitalzaron

10. ¿Interrumpió sus labores o tuvo que faltar al trabajo?

1. No
2. Sí

11. ¿Cuántas dosis de vacuna le han aplicado?

1. Una, solo la primera o la vacuna es de una sola dosis
2. Dos

12. ANTES DE LA VACUNACIÓN, ¿Consumió algún medicamento PARA PREVENIR los síntomas de la segunda dosis de vacuna?

1. No
2. Sí, ¿Cúal? __________

13. ¿Notó alguna diferencia de las molestias o síntomas entre la primera y segunda dosis de vacuna?

1. Me sentí mejor
2. Me sentí igual
3. Me sentí peor

14. Presentó alguna molestia o síntoma después de la segunda dosis de vacuna?

1. No
2. Sí

15. ¿Qué molestias o síntomas presentó o que molestias o síntomas se incrementaron?

1. Comezón en el sitio de aplicación
2. Enrojecimiento el sitio de aplicación
3. Hinchazón en el sitio de aplicación
4. Dolor en el sitio de aplicación
5. Dolor en el brazo
6. Dolor de cabeza
7. Dolor de garganta
8. Dolor de músculos
9. Dolor de huesos
10. Dolor en el pecho
11. Dolor de abdomen
12. Dolor al mover los ojos
13. Elevación de la presión arterial
14. Baja presión
15. Bochornos
16. Congestión nasal
17. Debilidad
18. Desmayo o pérdida del conocimiento
19. Diarrea
20. Escalofríos
21. Escurrimiento nasal
22. Falta de aire
23. Fatiga o cansancio
24. Exceso de sueño
25. Fiebre
26. Inflamación de cara o cuello
27. Inflamación de ganglios
28. Irritación de ojos
29. Malestar general
30. Manchas en la piel
31. Mareo
32. Náuseas
33. Palpitaciones o taquicardia
34. Ronchas
35. Sangrado
36. Sudores
37. Tos
38. Vómito
39. Otro, ¿Cuál? __________

Contagio con COVID-19

16. ¿Le han dicho que tuvo o tiene COVID-19?

1. No
2. Sí

17. ¿Cómo supo que tenía COVID-19 la primera o única vez que le dio?

1. Por prueba de PCR
2. Por prueba rápida de raspado nasal
3. Por prueba rápida en sangre
4. Solo por los síntomas, no me hicieron prueba

18. ¿Cómo considera que fue la intensidad de la enfermedad la primera o única vez que le dio?

1. Leve o sin síntomas
2. Moderado
3. Severo o grave
4. Muy grave, casi pierdo la vida

19. ¿Cuándo fue la primera o única vez que le dio COVID-19?

1. Antes de ponerse la vacuna
2. Después de la primera dosis o de la única dosis de vacuna
3. Después de la segunda dosis de vacuna

20.  La primera o única vez que le dio COVID-19, ¿Cuánto tiempo previo a la vacunación tuvo la enfermedad?

1. Menos de 40 días
2. 41-90 días
3. 91-180 días
4. Más de 180 días

 21. ¿Ha tenido COVID-19 más de una vez?

1. No
2. Sí

22. ¿Cómo supo que tenía COVID-19 la segunda vez?

1. Por PCR
2. por prueba rápida por raspado nasal
3. por prueba rápida en sangre
4. solo por los síntomas, no me hicieron prueba

23. ¿Cómo considera que fue la intensidad de la enfermedad la segunda vez?

1. Leve o sin síntomas
2. Moderado
3. Severo o grave
4. Muy grave, casi pierdo la vida

24. ¿Cuándo fue que tuvo COVID-19 por segunda vez?

1. Antes de ponerse la vacuna
2. Después de la primera dosis o de la única dosis de vacuna
3. Después de la segunda dosis

25.  La segunda vez que le dio COVID-19, ¿Cuánto tiempo previo a la vacunación tuvo la enfermedad?

1. Menos de 40 días
2. 41-90 días
3. 91-180 días
4. Más de 180 días

26. Después de vacunarse ¿le dijeron que tuvo o tiene COVID-19?

1. No
2. Sí

27. ¿Cómo supo que tenía COVID-19 después de vacunarse?

1. por PCR
2. por prueba rápida por raspado nasal
3. por prueba rápida en sangre
4. Solo por los síntomas, no me hicieron prueba

28. ¿Cómo considera que fue la intensidad de la enfermedad después de vacunarse?

1. Leve o sin síntomas
2. Moderado
3. Severo o grave
4. Muy grave, casi pierdo la vida

29. Sexo

1. Hombre
2. Mujer

Vacunación durante el embarazo y/o lactancia

30. ¿Estaba embarazada en el momento de la vacunación?

1. No
2. Sí

31. ¿Cuántas semanas de embarazo tenía?

|  |
| --- |

32. ¿Estaba lactando cuando la vacunaron?

1. No
2. Sí

33. Cuántos meses de edad tenía el infante al que le daba pecho?

|  |
| --- |

34. ¿Observó algún efecto de la vacuna en el infante?

1. No
2. Sí,  ¿cuál? __________

Enfermedades preexistentes y consumo de tabaco

35. ¿Padece de alguna otra enfermedad, diferente a COVID-19?

1. No
2. Sí

36. ¿Le han dicho que padece alguna de estas enfermedades?

1. Diabetes (azúcar en la sangre)
2. Presión alta (hipertensión)
3. Insuficiencia renal
4. Enfermedad pulmonar obstructiva crónica (EPOC)
5. Asma
6. Enfermedad inmunológica
7. Enfermedad cerebrovascular
8. Enfermedad cardiovascular
9. Enfermedad hepática
10. Otra, ¿Cuál? __________

37. ¿Es de las personas que sufre alergias?

1. No
2. Sí

38. ¿Estaba en tratamiento contra algún tipo de enfermedad durante la vacunación?

1. No
2. Sí, ¿Qué medicamentos? __________

39. ¿Es de las personas que fuma tabaco?

1. No
2. Sí

Información general

40. ¿Cuál considera que es su peso actual?


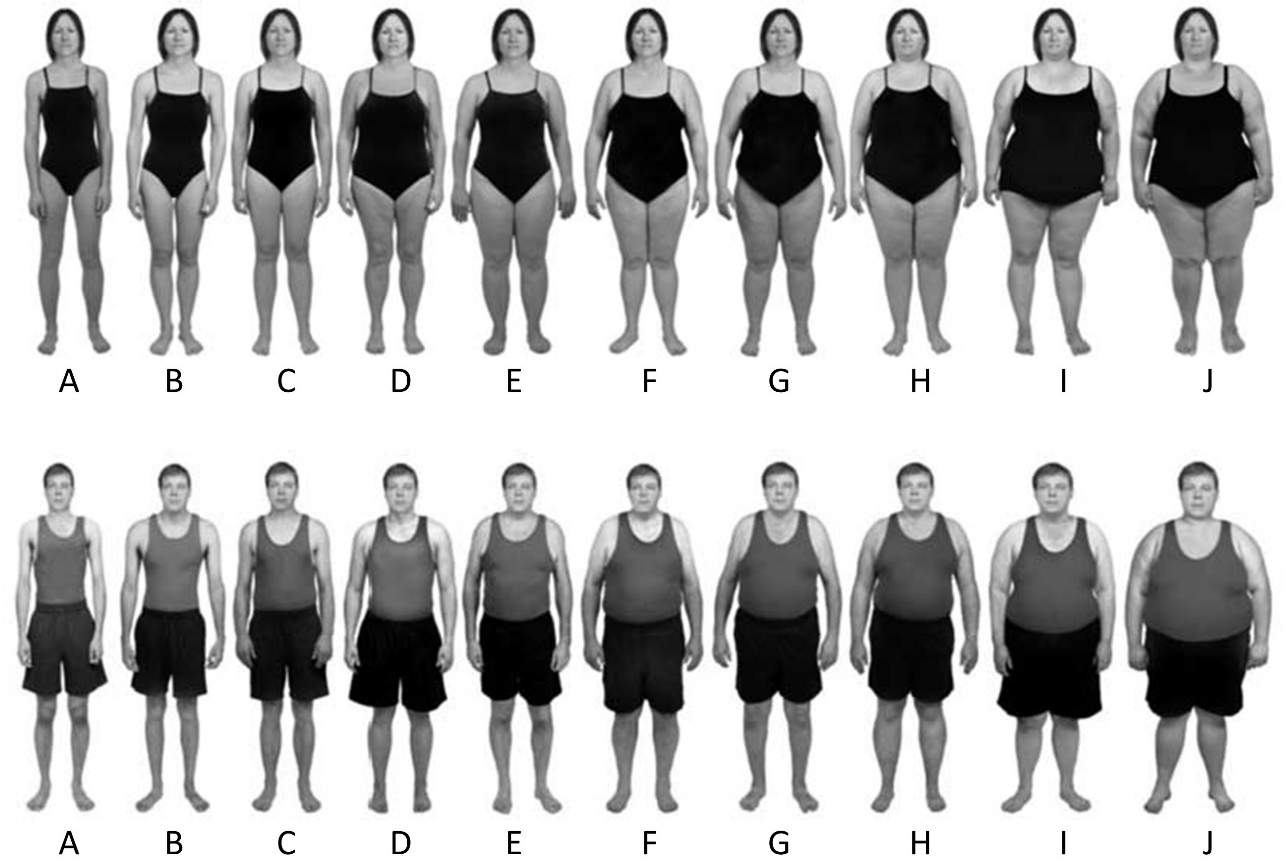


1. A
2. B
3. C
4. D
5. E
6. F
7. G
8. H
9. I
10. J

41. ¿Cuál es su peso actual en kilogramos?

1. No sé
2. peso en kilogramos __________

42. ¿Cuál es su altura actual en metros?

1. No sé
2. altura en metros __________

43. ¿Cuál es su grupo sanguíneo?

1. A&#43;
2. A-
3. B+
4. B-
5. AB+
6. AB-
7. O+
8. O-
9. No sabe / No se acuerda

44. ¿Cuántos años cumplidos tiene?

|  |
| --- |

45. ¿Hasta qué año de la escuela estudió?

1. Ninguno
2. Primaria
3. Secundaria
4. Preparatoria o carrera técnica
5. Licenciatura
6. Posgrado

46. ¿Cuál es su ocupación?

1. Ama de casa
2. Empleado
3. Autoempleado, trabaja por su cuenta
4. Pensionado o jubilado
5. Estudiante
6. Desempleado

47. ¿En qué estado vive?

1. Aguascalientes
2. Baja California
3. Baja California Sur
4. Campeche
5. Coahuila
6. Colima
7. Chiapas
8. Chihuahua
9. Ciudad de México
10. Durango
11. Guanajuato
12. Guerrero
13. Hidalgo
14. Jalisco
15. Estado de México
16. Michoacán
17. Morelos
18. Nayarit
19. Nuevo León
20. Oaxaca
21. Puebla
22. Querétaro
23. Quintana Roo
24. San Luis Potosí
25. Sinaloa
26. Sonora
27. Tabasco
28. Tamaulipas
29. Tlaxcala
30. Veracruz
31. Yucatán
32. Zacatecas
33. Vivo fuera de México

48. ¿En qué municipio vive?

|  |
| --- |
