# Supplementary material for: Extension and Severity of Self-Reported Side Effects of Seven COVID-19 Vaccines in Mexican Population
Source: Front Public Health. 2022 Mar 14;10:834744. doi: 10.3389/fpubh.2022.834744 (PMC8964147; doi:10.3389/fpubh.2022.834744)
Supplement: Supplementary file 2 [file Table_2.docx]

**Table S2.** Systemic side effects categorized by organ system and number of doses. Study on self-reported side effects of COVID-19 vaccines in the Mexican population, August - September 2021

| Side effect | First or single dose  (n = 4,024) | | Second dose  (n = 2,050) | |
| --- | --- | --- | --- | --- |
|  | n | % (95% confidence interval) | n | % (95% confidence interval) |
| General disorder |  |  |  |  |
| Lack of energy | 1,115 | 27.7 (26.3, 29.1) | 312 | 15.2 (13.7, 16.8) |
| Fatigue or tiredness | 1,107 | 27.5 (26.2, 28.9) | 477 | 23.3 (21.5, 25.1) |
| Fever | 926 | 23.0 (21.7, 24.3) | 236 | 11.5 (10.2, 13.0) |
| Chills | 817 | 20.3 (19.1, 21.6) | 214 | 10.4 (9.2, 11.8) |
| Malaise | 760 | 18.9 (17.7, 20.1) | 259 | 12.6 (11.3, 14.1) |
| Hot flashes | 255 | 6.3 (5.6, 7.1) | 60 | 2.9 (2.3, 3.7) |
| Sweating | 236 | 5.9 (5.2, 6.6) | 55 | 2.7 (2.1, 3.5) |
| Cardiovascular |  |  |  |  |
| Chest pain | 174 | 4.3 (3.7, 5.0) | 45 | 2.2 (1.6, 2.9) |
| A faster or lower heartbeat | 166 | 4.1 (3.6, 4.8) | 41 | 2 (1.5, 2.7) |
| Rise in blood pressure | 87 | 2.2 (1.8, 2.7) | 15 | 0.7 (0.4, 1.2) |
| Loss of blood pressure | 44 | 1.1 (0.8, 1.5) | 7 | 0.3 (0.2, 0.7) |
| Gastrointestinal |  |  |  |  |
| Nausea | 274 | 6.8 (6.1, 7.6) | 69 | 3.4 (2.7, 4.2) |
| Diarrhea | 177 | 4.4 (3.8, 5.1) | 71 | 3.5 (2.8, 4.3) |
| Abdominal pain | 96 | 2.4 (2.0, 2.9) | 25 | 1.2 (0.8, 1.8) |
| Vomiting | 57 | 1.4 (1.1, 1.8) | 20 | 1.0 (0.6, 1.5) |
| Musculoskeletal |  |  |  |  |
| Muscle pain | 1,293 | 32.1 (30.7, 33.6) | 370 | 18.0 (16.4, 19.8) |
| Bone or joint pain | 713 | 17.7 (16.6, 18.9) | 222 | 10.8 (9.6, 12.2) |
| Nervous system disorders |  |  |  |  |
| Headache | 1,537 | 38.2 (36.7, 39.7) | 541 | 26.4 (24.5, 28.3) |
| Desire to sleep | 912 | 22.7 (21.4, 24.0) | 300 | 14.6 (13.2, 16.2) |
| Dizziness and giddiness | 265 | 6.6 (5.9, 7.4) | 76 | 3.7 (3.0, 4.6) |
| Respiratory |  |  |  |  |
| Stuffy nose | 197 | 4.9 (4.3, 5.6) | 61 | 3 (2.3, 3.8) |
| Running nose | 124 | 3.1 (2.6, 3.7) | 54 | 2.6 (2, 3.4) |
| Difficulty breathing (dyspnea) | 107 | 2.7 (2.2, 3.2) | 25 | 1.2 (0.8, 1.8) |
| Cough | 90 | 2.2 (1.8, 2.7) | 34 | 1.7 (1.2, 2.3) |
| Skin and subcutaneous tissue |  |  |  |  |
| Irritated eyes | 130 | 3.2 (2.7, 3.8) | 31 | 1.5 (1.1, 2.1) |
| Rash / hives / irritable skin | 50 | 1.2 (0.9, 1.6) | 11 | 0.5 (0.3, 1.0) |
| Blood and lymphatic |  |  |  |  |
| Lymph nodes tenderness | 91 | 2.3 (1.8, 2.8) | 38 | 1.9 (1.4, 2.5) |
| Other |  |  |  |  |
| Sore throat | 191 | 4.7 (4.1, 5.4) | 72 | 3.5 (2.8, 4.4) |
| Eye movement pain | 253 | 6.3 (5.6, 7.1) | 55 | 2.7 (2.1, 3.5) |
