# Supplementary material for: Extension and Severity of Self-Reported Side Effects of Seven COVID-19 Vaccines in Mexican Population
Source: Front Public Health. 2022 Mar 14;10:834744. doi: 10.3389/fpubh.2022.834744 (PMC8964147; doi:10.3389/fpubh.2022.834744)
Supplement: Supplementary file 3 [file Table_3.docx]

**Table S3.** Side effects after first dose categorized by organ system and type of vaccine. Study on self-reported side effects of COVID-19 vaccines in the Mexican population, August - September 2021

|  | Pfizer^1^  (n = 4,024) | AstraZeneca^2^ (n =1,579) | Moderna^3^  (n =1,193) | SinoVac^4^  (n =52) | Sputnik V^5^  (n =299) | Cansino^6^  (n =202) | J & J^7^  (n =598) | Chi-square p value |
| --- | --- | --- | --- | --- | --- | --- | --- | --- |
|  | n (%) | n (%) | n (%) | n (%) | n (%) | n (%) | n (%) |  |
| **Local side effect** |  |  |  |  |  |  |  |  |
| Arm /injection site pain | 1076 (68.1%) | 867 (72.7%) | 34 (65.4%) | 135 (45.2%) | 143 (70.8%) | 339 (56.7%) | 52 (53.6%) | 0.0001 |
| Injection site swelling | 134 (8.5%) | 111 (9.3%) | 10 (19.2%) | 8 (2.7%) | 23 (11.4%) | 41 (6.9%) | 6 (6.2%) | 0.0001 |
| Injection site itching | 51 (3.2%) | 82 (6.9%) | 4 (7.7%) | 8 (2.7%) | 13 (6.4%) | 48 (8.0%) | 4 (4.1%) | 0.0001 |
| Injection site redness | 54 (3.4%) | 60 (5.0%) | 7 (13.5%) | 1 (0.3%) | 11 (5.4%) | 29 (4.8%) | 4 (4.1%) | 0.0001 |
| **Systemic side effect** |  |  |  |  |  |  |  |  |
| General disorder |  |  |  |  |  |  |  |  |
| Hot flashes | 44 (2.8%) | 146 (12.2%) | 2 (3.8%) | 9 (3.0%) | 16 (7.9%) | 36 (6.0%) | 2 (2.1%) | 0.0001 |
| Lack of energy | 286 (18.1%) | 481 (40.3%) | 8 (15.4%) | 37 (12.4%) | 72 (35.6%) | 202 (33.8%) | 27 (27.8%) | 0.0001 |
| Chills | 139 (8.8%) | 439 (36.8%) | 5 (9.6%) | 10 (3.3%) | 64 (31.7%) | 129 (21.6%) | 29 (29.9%) | 0.0001 |
| Fatigue or tiredness | 289 (18.3%) | 458 (38.4%) | 10 (19.2%) | 40 (13.4%) | 74 (36.6%) | 206 (34.4%) | 28 (28.9%) | 0.0001 |
| Fever | 155 (9.8%) | 515 (43.2%) | 6 (11.5%) | 14 (4.7%) | 67 (33.2%) | 142 (23.7%) | 26 (26.8%) | 0.0001 |
| Malaise | 171 (10.8%) | 358 (30.0%) | 5 (9.6%) | 17 (5.7%) | 59 (29.2%) | 129 (21.6%) | 20 (20.6%) | 0.0001 |
| Sweating | 29 (1.8%) | 145 (12.2%) | 2 (3.8%) | 6 (2.0%) | 16 (7.9%) | 30 (5.0%) | 8 (8.2%) | 0.0001 |
| Cardiovascular |  |  |  |  |  |  |  |  |
| Chest pain | 43 (2.7%) | 89 (7.5%) | 0 (0.0%) | 9 (3.0%) | 11 (5.4%) | 22 (3.7%) | 0 (0.0%) | 0.0001 |
| Rise in blood pressure | 29 (1.8%) | 38 (3.2%) | 0 (0.0%) | 5 (1.7%) | 2 (1.0%) | 13 (2.2%) | 0 (0.0%) | ^a^ |
| Loss of blood pressure | 5 (0.3%) | 18 (1.5%) | 1 (1.9%) | 0 (0.0%) | 4 (2.0%) | 12 (2.0%) | 4 (4.1%) | ^a^ |
| A faster or lower heartbeat | 53 (3.4%) | 81 (6.8%) | 0 (0.0%) | 3 (1.0%) | 8 (4.0%) | 19 (3.2%) | 2 (2.1%) | 0.0001 |
| Gastrointestinal |  |  |  |  |  |  |  |  |
| Abdominal pain | 14 (0.9%) | 49 (4.1%) | 0 (0.0%) | 6 (2.0%) | 9 (4.5%) | 13 (2.2%) | 5 (5.2%) | ^a^ |
| Diarrhea | 45 (2.8%) | 82 (6.9%) | 1 (1.9%) | 13 (4.3%) | 7 (3.5%) | 27 (4.5%) | 2 (2.1%) | 0.0001 |
| Nausea | 39 (2.5%) | 161 (13.5%) | 3 (5.8%) | 13 (4.3%) | 13 (6.4%) | 40 (6.7%) | 5 (5.2%) | 0.0001 |
| Vomiting | 10 (0.6%) | 32 (2.7%) | 1 (1.9%) | 2 (0.7%) | 2 (1.0%) | 10 (1.7%) | 0 (0.0%) | ^a^ |
| Musculoskeletal and connective tissue disorders | |  |  |  |  |  |  |  |
| Muscle pain | 299 (18.9%) | 617 (51.7%) | 6 (11.5%) | 38 (12.7%) | 82 (40.6%) | 212 (35.5%) | 36 (37.1%) | 0.0001 |
| Bone or joint pain | 168 (10.6%) | 334 (28.0%) | 5 (9.6%) | 19 (6.4%) | 47 (23.3%) | 124 (20.7%) | 15 (15.5%) | 0.0001 |
| Nervous system disorders |  |  |  |  |  |  |  |  |
| Headache | 443 (28.1%) | 654 (54.8%) | 9 (17.3%) | 60 (20.1%) | 95 (47.0%) | 237 (39.6%) | 36 (37.1%) | 0.0001 |
| Desire to sleep | 276 (17.5%) | 327 (27.4%) | 8 (15.4%) | 52 (17.4%) | 52 (25.7%) | 172 (28.8%) | 23 (23.7%) | 0.0001 |
| Dizziness and giddiness | 59 (3.7%) | 138 (11.6%) | 2 (3.8%) | 10 (3.3%) | 10 (5.0%) | 42 (7.0%) | 4 (4.1%) | 0.0001 |
| Respiratory |  |  |  |  |  |  |  |  |
| Stuffy nose | 43 (2.7%) | 93 (7.8%) | 1 (1.9%) | 20 (6.7%) | 14 (6.9%) | 23 (3.8%) | 3 (3.1%) | 0.0001 |
| Running nose | 29 (1.8%) | 59 (4.9%) | 0 (0.0%) | 13 (4.3%) | 8 (4.0%) | 14 (2.3%) | 1 (1.0%) | 0.0001 |
| Difficulty breathing (dyspnea) | 21 (1.3%) | 61 (5.1%) | 0 (0.0%) | 2 (0.7%) | 10 (5.0%) | 12 (2.0%) | 1 (1.0%) | 0.0001 |
| Cough | 19 (1.2%) | 42 (3.5%) | 0 (0.0%) | 10 (3.3%) | 7 (3.5%) | 12 (2.0%) | 0 (0.0%) | ^a^ |
| Skin and subcutaneous tissue disorders | |  |  |  |  |  |  |  |
| Irritated eyes | 22 (1.4%) | 67 (5.6%) | 1 (1.9%) | 8 (2.7%) | 9 (4.5%) | 21 (3.5%) | 2 (2.1%) | 0.0001 |
| Skin rash or hives/irritable skin | 7 (0.4%) | 17 (1.4%) | 0 (0.0%) | 3 (1.0%) | 1 (0.5%) | 4 (0.7%) | 1 (1.0%) | ^a^ |
| Blood and lymphatic system disorders | |  |  |  |  |  |  |  |
| Lymph nodes tenderness | 27 (1.7%) | 39 (3.3%) | 0 (0.0%) | 6 (2.0%) | 5 (2.5%) | 13 (2.2%) | 1 (1.0%) | ^a^ |
| Other |  |  |  |  |  |  |  |  |
| Sore throat | 42 (2.7%) | 92 (7.7%) | 2 (3.8%) | 13 (4.3%) | 14 (6.9%) | 24 (4.0%) | 4 (4.1%) | ^a^ |
| Eye movement pain | 45 (2.8%) | 140 (11.7%) | 0 (0.0%) | 7 (2.3%) | 17 (8.4%) | 35 (5.9%) | 9 (9.3%) | 0.0001 |

^1^ BNT162b2 (Pfizer-BioNTech), ^2^ ChAdOx1 (AstraZeneca–Oxford), ^3^ ARNm-1273 (Moderna, Inc.), ^4^ CoronaVac (Sinovac Life Sciences), ^5^ Gam-COVID-Vac (Gamaleya's Sputnik V), ^6^Ad5-nCoV (CanSinoBIO), and ^7^ Ad26.CoV2.S (Johnson & Johnson/Janssen). ^a^ Cannot be interpreted; no expected frequency should be less than 1 and not over 20% of expected frequencies should be less than 5
