# Supplementary material for: Extension and Severity of Self-Reported Side Effects of Seven COVID-19 Vaccines in Mexican Population
Source: Front Public Health. 2022 Mar 14;10:834744. doi: 10.3389/fpubh.2022.834744 (PMC8964147; doi:10.3389/fpubh.2022.834744)
Supplement: Supplementary file 4 [file Table_4.docx]

**Table S4.** Side effects after second dose categorized by organ system and type of vaccine. Study on self-reported side effects of COVID-19 vaccines in the Mexican population, August - September 2021

|  | Pfizer^1^  (n =1378) | AstraZeneca^2^  (n =338) | Moderna^3^  (n =50) | SinoVac^4^  (n =177) | Sputnik V^5^  (n =71) | Chi-square p value |
| --- | --- | --- | --- | --- | --- | --- |
|  | n (%) | n (%) | n (%) | n (%) | n (%) |  |
| **Local side effect** |  |  |  |  |  |  |
| Arm /injection site pain | 786 (57.0%) | 111 (32.8%) | 38 (76.0%) | 67 (37.9%) | 37 (52.1%) | 0.0001 |
| Injection site swelling | 1302 (5.5%) | 327 (3.3%) | 43 (14.0%) | 173 (2.3%) | 69 (2.8%) | ^a^ |
| Injection site itching | 42 (3.0%) | 5 (1.5%) | 3 (6.0%) | 1 (0.6%) | 1 (1.4%) | ^a^ |
| Injection site redness | 35 (2.5%) | 5 (1.5%) | 4 (8.0%) | 3 (1.7%) | 0 (0.0%) | ^a^ |
| **Systemic side effect** |  |  |  |  |  |  |
| General disorder |  |  |  |  |  |  |
| Hot flashes | 42 (3.0%) | 13 (3.8%) | 3 (6.0%) | 1 (0.6%) | 0 (0.0%) | ^a^ |
| Lack of energy | 233 (16.9%) | 27 (8.0%) | 10 (20.0%) | 18 (10.2%) | 12 (16.9%) | 0.0001 |
| Chills | 150 (10.9%) | 25 (7.4%) | 17 (34.0%) | 6 (3.4%) | 8 (11.3%) | 0.0001 |
| Fatigue or tiredness | 353 (25.6%) | 46 (13.6%) | 23 (46.0%) | 26 (14.7%) | 17 (23.9%) | 0.0001 |
| Fever | 175 (12.7%) | 26 (7.7%) | 14 (28.0%) | 5 (2.8%) | 6 (8.5%) | 0.0001 |
| Malaise | 188 (13.6%) | 32 (9.5%) | 12 (24.0%) | 9 (5.1%) | 10 (14.1%) | 0.001 |
| Sweating | 36 (2.6%) | 10 (3.0%) | 5 (10.0%) | 2 (1.1%) | 1 (1.4%) | 0.014 |
| Cardiovascular |  |  |  |  |  |  |
| Chest pain | 38 (2.8%) | 5 (1.5%) | 0 (0.0%) | 0 (0.0%) | 0 (0.0%) | ^a^ |
| Rise in blood pressure | 12 (0.9%) | 1 (0.3%) | 1 (2.0%) | 1 (0.6%) | 0 (0.0%) | ^a^ |
| Loss of blood pressure | 4 (0.3%) | 2 (0.6%) | 1 (2.0%) | 0 (0.0%) | 0 (0.0%) | ^a^ |
| A faster or lower heartbeat | 32 (2.3%) | 7 (2.1%) | 1 (2.0%) | 0 (0.0%) | 0 (0.0%) | ^a^ |
| Gastrointestinal |  |  |  |  |  |  |
| Abdominal pain | 17 (1.2%) | 6 (1.8%) | 0 (0.0%) | 0 (0.0%) | 1 (1.4%) | ^a^ |
| Diarrhea | 43 (3.1%) | 12 (3.6%) | 2 (4.0%) | 5 (2.8%) | 9 (12.7%) | ^a^ |
| Nausea | 47 (3.4%) | 14 (4.1%) | 2 (4.0%) | 4 (2.3%) | 1 (1.4%) | ^a^ |
| Vomiting | 12 (0.9%) | 4 (1.2%) | 2 (4.0%) | 1 (0.6%) | 0 (0.0%) | ^a^ |
| Musculoskeletal and connective tissue disorders | |  |  |  |  |  |
| Muscle pain | 271 (19.7%) | 44 (13.0%) | 16 (32.0%) | 16 (9.0%) | 13 (18.3%) | 0.000 |
| Bone or joint pain | 163 (11.8%) | 24 (7.1%) | 9 (18.0%) | 8 (4.5%) | 10 (14.1%) | 0.002 |
| Nervous system disorders |  |  |  |  |  |  |
| Headache | 386 (28.0%) | 75 (22.2%) | 20 (40.0%) | 28 (15.8%) | 18 (25.4%) | 0.0001 |
| Desire to sleep | 219 (15.9%) | 27 (8.0%) | 9 (18.0%) | 24 (13.6%) | 11 (15.5%) | 0.006 |
| Dizziness and giddiness | 52 (3.8%) | 16 (4.7%) | 0 (0.0%) | 4 (2.3%) | 1 (1.4%) | ^a^ |
| Respiratory |  |  |  |  |  |  |
| Stuffy nose | 42 (3.0%) | 8 (2.4%) | 0 (0.0%) | 4 (2.3%) | 4 (5.6%) | ^a^ |
| Running nose | 42 (3.0%) | 5 (1.5%) | 0 (0.0%) | 3 (1.7%) | 3 (4.2%) | ^a^ |
| Difficulty breathing (dyspnea) | 20 (1.5%) | 3 (0.9%) | 0 (0.0%) | 1 (0.6%) | 1 (1.4%) | ^a^ |
| Cough | 24 (1.7%) | 4 (1.2%) | 0 (0.0%) | 2 (1.1%) | 3 (4.2%) | ^a^ |
| Skin and subcutaneous tissue disorders | |  |  |  |  |  |
| Irritated eyes | 23 (1.7%) | 3 (0.9%) | 1 (2.0%) | 3 (1.7%) | 1 (1.4%) | ^a^ |
| Skin rash or hives/irritable skin | 4 (0.3%) | 0 (0.0%) | 0 (0.0%) | 2 (1.1%) | 1 (1.4%) | ^a^ |
| Blood and lymphatic system disorders | |  |  |  |  |  |
| Lymph nodes tenderness | 32 (2.3%) | 2 (0.6%) | 3 (6.0%) | 1 (0.6%) | 0 (0.0%) | ^a^ |
| Other |  |  |  |  |  |  |
| Sore throat | 49 (3.6%) | 12 (3.6%) | 2 (4.0%) | 3 (1.7%) | 3 (4.2%) | ^a^ |
| Eye movement pain | 36 (2.6%) | 9 (2.7%) | 1 (2.0%) | 5 (2.8%) | 2 (2.8%) | ^a^ |

^1^ BNT162b2 (Pfizer-BioNTech), ^2^ ChAdOx1 (AstraZeneca–Oxford), ^3^ ARNm-1273 (Moderna, Inc.), ^4^ CoronaVac (Sinovac Life Sciences), ^5^ Gam-COVID-Vac (Gamaleya's Sputnik V). ^a^ Cannot be interpreted; no expected frequency should be less than 1 and not over 20% of expected frequencies should be less than 5
